# Treatment of recalcitrant vitiligo by autologous non-cultured and trypsinized melanocyte grafting in the west of Iran^☆^

**DOI:** 10.1016/j.abd.2021.08.003

**Published:** 2022-02-17

**Authors:** Iraj Ghorbani, Mozafar Khazaei, Hossein Kavoussi, Ali Ebrahimi, Mansour Rezaei, Reza Kavoussi, Kamran Mansouri

**Affiliations:** a Dermatology Department, Hajdaie Dermatology Clinic, Students Research Committee, Medicine School, Kermanshah University of Medical Sciences, Kermanshah, Iran; b Fertility and Infertility Research Center, Health Technology Institute, Kermanshah University of Medical Sciences, Kermanshah, Iran; c Dermatology Department, Hajdaie Dermatology Clinic, Medicine School, Kermanshah University of Medical Sciences (KUMS), Kermanshah, Iran; d Health School, Family Health Research Center of Kermanshah University of Medical Sciences, Kermanshah, Iran; e Medicine School, Kermanshah University of Medical Sciences, Kermanshah, Iran; f Medical Biology Research Center, Health Technology Institute, Kermanshah University of Medical Sciences, Kermanshah, Iran

**Keywords:** Autoantigens, Melanocytes, Vitiligo

## Abstract

**Background:**

Vitiligo is a common disease with a high burden, and its recalcitrant type is unresponsive to current medical treatments. Autologous non-cultured and trypsinized melanocyte grafting, which is a simple and experience-based procedure, has been suggested for the treatment of vitiligo.

**Objective:**

To assess autologous non-cultured and trypsinised melanocyte grafting in recalcitrant vitiligo.

**Methods:**

This clinical trial was done on 28 patients (20 females and 8 males). After demarcation and preparation of both donor and recipient sites, both sites were shaved by a curette. The materials harvested from the donor site were trypsinized and centrifuged. The resulting suspension was mixed with hyaluronic acid gel and was spread over the shaved recipient area.

**Results:**

Twenty-eight patients with a total of 108 lesions and a mean age of 25.93 ± 7.11 years were included in the present study. Generalized vitiligo (57.1%) was the most common clinical type and the face and neck regions (38%) were the most frequent treated sites. Good to excellent repigmentation was seen in the face and neck, trunk, upper extremity, and genitals in 31 (57.4%), 11 (20.4%), 9 (16.7%) and 3 (5.5%) patients, respectively. Face and neck showed significantly better results (p < 0.05).

**Study limitations:**

Low sample size and single-center study.

**Conclusion:**

Autologous non-cultured and trypsinized melanocyte grafting is a safe method with satisfactory outcomes in recalcitrant vitiligo. Appropriate training of physicians and proper use of specialists’ experiences can be effective in increasing the improvement rate.

## Introduction

Vitiligo is a common pigmenting dermatosis that is associated with problems in several contexts such as education, family, marriage, employment, sex, and social relations.^1^, ^2^, ^3^, ^4^

There is no definitive cure for vitiligo; therefore, many surgical options such as autologous cultured or non-cultured melanocyte grafting have been developed for the treatment of vitiligo in recent years.^3^, ^5^, ^6^, ^7^, ^8^, ^9^, ^10^, ^11^, ^12^, ^13^

In Recalcitrant Vitiligo (RV), including vitiligo in hair-bearing areas with leukotrichia or in glabrous areas, the disease is resistant to medical treatment and requires surgical options interventions such as Autologous Non-cultured and Trypsinised Melanocyte Grafting (ANTMG).^5^, ^7^, ^9^, ^14^, ^15^, ^16^, ^17^, ^18^

Due to the high prevalence of vitiligo many psychosocial problems with high treatment costs, the authors used ANTMG for the treatment of RV.

## Materials and methods

### Study design and population

This interventional and follow-up study was performed on 31 patients over a period of 18 months from 2019 to 2020 at Hajdaie dermatology referral clinic of Kermanshah University of Medical Sciences, Iran. ANTMG procedure and follow-up were performed in 28 out of 31 patients; 3 patients were excluded from the study. Patients were selected from among those who were referred to the present study’s clinic.

The authors recruited vitiligo patients who were clinically documented by two dermatologists who were not involved in the present study. Further, the patients should not have a new or enlarged vitiliginous lesion at least in the past six months, having at least three patches, vitiliginous lesions less than 100 square centimeters, lesions in glabrous areas or in non-glabrous areas with leukotrichia, unresponsiveness to the current treatment over the past two years. The breastfeeding and pregnant women, patients lower than 15 or more than 60 years of age, and patients with a positive history of Koebner phenomena keloid or a hypertrophic scar, immunodeficiency, psychologic disorders, active infection in the recipient or donor sites, and unstable and universal vitiligo were excluded from the study.

Comprehensive information about ANTMG was given to all patients. Then, written consent was obtained from the patients, and they were finally enrolled in the study. The current study was approved by the Ethics Committee of Kermanshah University of Medical Science and registered in the Iranian Registration Clinical Trial (IRCT) database (IRCT20130812014333N146). The participants’ information was kept confidential.

### Procedure

**Figure 1:**
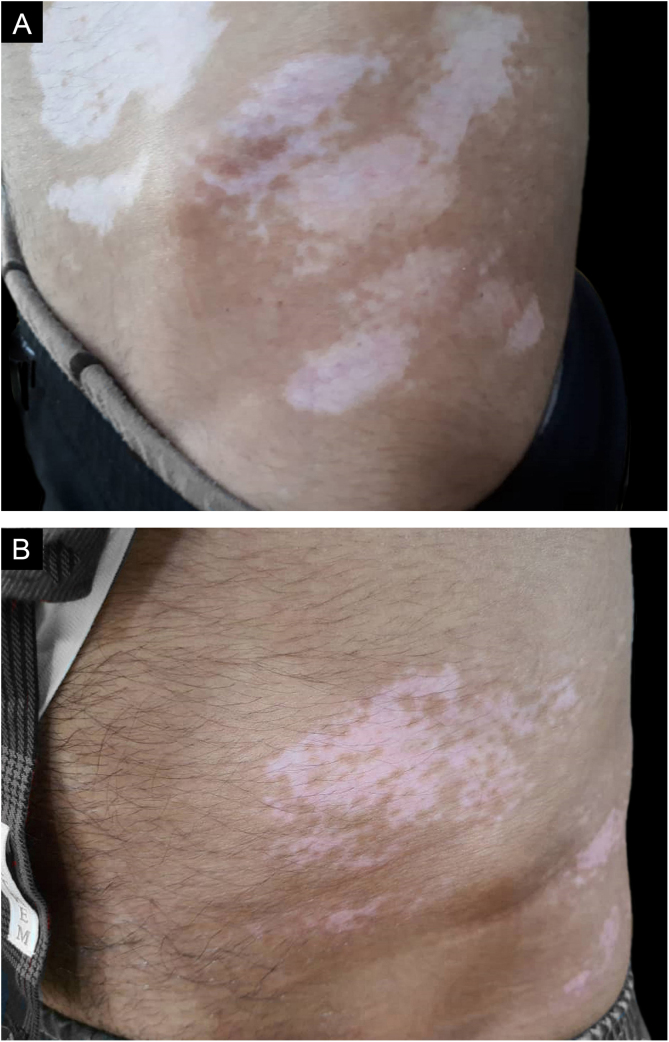
(A), A male patient with several depigmented patches in the trunk. (B), Treatment outcome with more than 50% repigmentation rate after 18 months.

**Figure 2:**
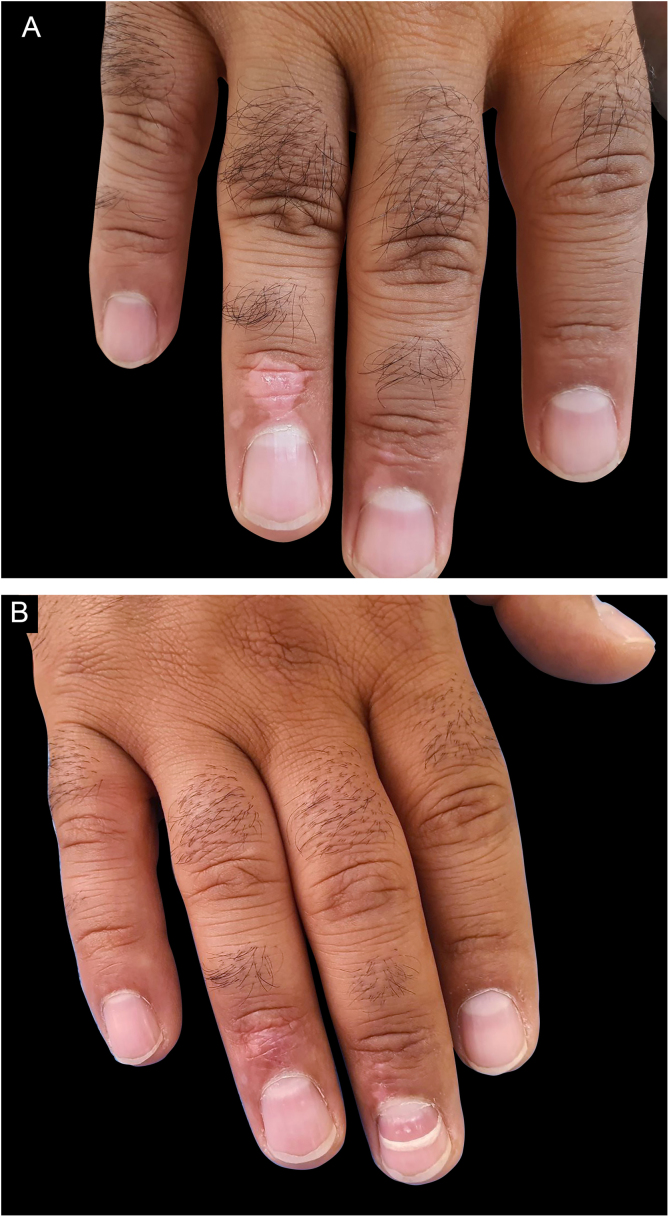
(A), A male patient with vitiliginous patches in the acral area. (B), Treatment result with excellent outcome after 18 months.

The authors chose the lateral areas of the thighs or buttocks with normal skin color as the donor sites. The area of the donor site was considered a quarter of the recipient site.(Figs. 1A, 1B, 2A and 2B)

The selected donor site was demarcated and anesthetized by injection of 2% lidocaine without epinephrine. Then, a disposable and sharp curette nº 2 was used in order to harvest the autologous materials. The collected sample was transferred to a container and ground by bistoury blade nº10. Next, the harvested material was incubated with trypsin at a very low concentration (0.25%) for 10 minutes at 37 °C. After that, for its inhibition, 100 µL of patient serum was used and was then washed with normal saline.

To obtain an appropriate suspension, the processed solution was centrifuged. The sample of the resulting material was prepared as a direct smear and evaluated with a light microscope to demonstrate the presence of melanocytes.

Shaving the recipient depigmented patches was done in a similar manner as in the donor sites employing the local injection of lidocaine and a curate device that as used in donor sites. The harvested processed materials were mixed with Revofil hyaluronic acid product (23 mg/mL) in a ratio of 5 to 1 and were spread evenly on the recipient areas. The recipient site was dressed with a semi-permeable dressing (Tegaderm), and the donor site was dressed with gauze and tetracycline ointment. The patient was prescribed oral antibiotics (cephalexin 500 mg every 6 hours) after the procedure for one week. The patient was also advised to immobilize the recipient site for a week. The donor site dressing was changed daily by the patient, but the recipient site dressing was changed and replaced by the researcher after a week.

The patient was visited in the first and third weeks, the second month, and then every two months for 18 months. The Repigmentation rate of the treated areas was calculated over 18 months, where repigmentation was stationary in all patients, based on the pre-and post-operation photographs by two dermatologists that were not involved in this study. The percentage of repigmentation and possible complications were recorded in the patients’ files.

Repigmentation was graded as excellent (more than 75%), good (50%–74%), moderate (25%–49%), and poor (less than 25%) pigmentation of the treated regions.

### Statistical analysis

The gathered data were analyzed by SPSS V22. To compare the qualitative variables in two groups, chi-square or Fisher’s exact test was used, and p < 0.05 was considered significant.

## Results

Out of 28 patients, 20 (71.4%) were female, and 8 (28.6) were male, with a mean age of 25.93 ± 7.11 years and 108 vitiliginous patches.

The range of disease duration was 2–12 years, with a mean duration of 5.14 ± 3.2 years. Of patients, 12 (42.9%) had a family history of vitiligo disease (Table 1).

**Table 1:** Demographic and clinical characteristics of our patients.

Variables	
Number of patients (n)	28
Number of vitiliginous patches, n (%)	108
Mean age of patients (years)	25.93 ± 7.11
Sex	
Female, n (%)	20 (71.4%)
Male	8 (28.6%)
Familial history, n (%)	
Positive	12 (42.9%)
Negative	16 (57.1%)
Mean duration of disease (years)	5.14 ± 3.2
Type of vitiligo, n(%)	
Focal	4 (14.3%)
Segmental	8 (28.6%)
Acrofacial	4 (14.3%)
Vulgaris	12 (42.8%)
Involved sites, n (%)	
Upper extremity	17(60.7%)
Face and neck	17(60.7%)
Trunk	12 (42.8%)
Lower extremity	8 (28.6%)
Genital area	2 (7.1%)
Treated sites, n (%)	
Face and neck	41(38.0%)
Upper extremity	19 (17.6%)
Trunk	37 (34.2%)
Genital area	11 (10.2%)

Regarding the type of vitiligo, 19 (57.1%) patients, 4 (14.3%) acrofacial and 12 (42.9%) vulgaris, had the generalized type, and 12 (42.9%) patients, 4 (14.3%) focal and 8 (28.6%) segmental, had the localized type (Table 1).

For better statistical analysis and a more accurate assessment of the correlation between variables, the authors classified the involved and treated areas into the trunk, face and neck, and upper and lower extremities. The repigmentation outcome was also classified into poor to moderate and good to excellent.

The frequencies of the involved and treated sites are shown in Table 1.

The frequencies of the treated sites with more details included the fingers 15 (13.9%), chest 12 (11.1%), genitals 11 (10.2%), and cheeks 9 (8.3%). The other treated areas are listed in Table 2.

**Table 2:** Treated sites and outcomes of repigmentation in our patients.

Treated sites	Frequency	**Outcome of repigmentation**		
		Good to excellent	Poor to moderate	p-value
Upper extremity Fingers, n (%)	15 (78.9%)	7 (46.7%)	8 (53.3%)	
Dorsal of hand	3 (15.8%)	2 (66.7%)	1 (33.3%)	
Forearm	1 (5.3%)	0 (0.0%)	1 (100.0%)	
Total	19 (100.0%)	9 (47.4%)	10 (52.6%)	
Face and neck, n (%)				
Cheek	9 (21.9%)	7 (77.8%)	2 (22.2%)	
Lower lid	8 (19.5%)	5 (62.5%)	3 (37.5%)	
Forehead	6 (14.6%)	6 (100.0%)	0 (0.0%)	
Neck	6 (14.6%)	3 (50.0%)	3 (50.0%)	
Chin	3 (7.3%)	2 (66.7%)	1 (33.3%)	
Upper lid	3 (7.3%)	2 (66.7%)	1 (33.3%)	0.001
Upper lip	2 (4.9%)	2 (100.0%)	0 (0.0%)	
Temple	2 (4.9%)	2 (100.0%)	0 (0.0%)	
Lateral face	2 (4.9%)	2 (100.0%)	0 (0.0%)	
Total	41 (100%)	31(75.6%)	10 (24.4%)	
Trunk, n (%)				
Chest	12 (32.4%)	2 (16.7%)	10(83.3%)	
Flank	8 (21.6%)	2 (25.0%)	6 (75.0%)	
Abdomen	7 (18.9%)	3 (42.9%)	4 (57.1%)	
Back	6 (16.2%)	2 (33.3%)	4 (66.7%)	
Shoulder	4 (10.8%)	2 (50.0%)	2 (50.0%)	
Total	37 (100%)	11 (29.7%)	26 (70.3%)	
Genital areas, n (%)	11 (100.0%)	3 (27.3%)	8 (72.7%)	
Total	11 (100.0%)	3 (27.3%)	8 (72.7%)	
All treated areas	108(100.0%)	54(50.0%)	54 (50.0.0%)	

The outcome of skin repigmentation was good to excellent in 54 (50.0%) and poor to moderate in 54 (50.0%) treated patches, respectively (Table 2).

Good to excellent repigmentation of vitiliginous patches in the face and neck, trunk, upper extremity, and genitals were seen in 31 (57.4%), 11 (20.4%), 9 (16.7%), and 3 (5.5%) patients, respectively (Table 2).

Poor to moderate repigmentation of vitiliginous patches in the face and neck, trunk, upper extremity, and genitals were seen in 10 (18.5%), 26 (48.1%), 10 (18.5%), and 8 (14.8%) patients, respectively (Table 2).

There was a significant relationship between the outcome of repigmentation and the treated site (p > 0.05). Face and neck showed significantly better repigmentation than the other area (p < 0.05) (Table 2).

Pruritus, burning, erythema, post-inflammatory hyperpigmentation, and vesicle as complications of the current study were seen in 24 (22.2%), 21 (19.4%), 14 (13.0%), 10 (9.2%), and 4 (3.7%) treated sites respectively. All of these complications were transient and improved after two months.

Koebner phenomenon, dyschromia, and unsightly scars were not found in any treatment or donor sites.

## Discussion

The present study’s findings revealed ANTMG was an efficacious, simple, and safe procedure for the treatment of vitiligo, especially the recalcitrant form. ANTMG also showed a significantly better treatment result in the face and neck.

Most of the patients, with a median age of 25.93 ± 7.11 years, were female (71.4%), had generalized vitiligo type (57.1%), and were subjected to ANTMG commonly in the face and neck (38%).

In the present study, consistent with most studies,^12^, ^17^, ^18^, ^19^, ^20^, ^21^ there was no significant difference between the treatment outcome of vitiligo patches through ANTMG and age, disease duration, and sex.

Although the limited type of vitiligo has shown a significantly better response to the generalized type by ANTMG,^6^, ^10^, ^18^ in the study’s patients, both clinical types of vitiligo indicated no important difference in treatment outcome. The authors think it is highly important to select the vitiligo patients properly and perform the procedure accurately to achieve acceptable treatment results, even in any clinical type of vitiligo.

Studies have reported variable results for ANTMG in the treatment of vitiligo, ranging from poor to excellent outcomes.^5^, ^6^, ^7^, ^8^, ^9^, ^10^, ^11^, ^12^, ^13^, ^14^, ^15^, ^16^, ^17^, ^18^ Expertise in ANTGM procedure is an important factor in achieving a satisfactory treatment result. In this procedure, the use of special medical instruments, the method of harvesting materials at the donor sites, preparation of the recipient site, and adjuvant therapies after an operation can affect the treatment outcome.^6^, ^7^, ^13^, ^14^^,^^18^

The site of vitiligo patches, type of vitiligo, and disease stability and recalcitrance are other important factors involved in obtaining an acceptable outcome.^5^, ^6^, ^9^, ^10^^,^^12^, ^14^, ^18^

The present study’s findings revealed 50% of the treated vitiliginous sites showed good to excellent outcomes, which is a nearly acceptable treatment result. The advantages of the present study were that all of the patients had a resistant form of the disease, and most of the patients had a generalized form of vitiligo and difficult-treated areas such as acral and genital sites, which could have negatively affected the treatment outcomes.

In line with most studies,^6^, ^10^, ^13^, ^18^ our patients had some complaints such as pruritus and PIH in the recipient site after the procedure, but all of them were transient and improved after several weeks.

One of the present study’s main concerns was the possible occurrence of depigmentation, i.e., the Koebner phenomenon at the donor site, which was observed in some studies. However, it was not observed in any of the donor sites of our patients, indicating the proper selection of patients and stability of vitiligo in the patients.

## Conclusion

With proper selection of vitiligo patients, ANTMG is a safe and low-price procedure with a satisfactory outcome in recalcitrant vitiligo. Appropriate training of physicians and proper use of the specialists’ experiences can be effective in increasing the improvement rate. Future studies are suggested to be carried out in several centers with more cases and more variables.

## Financial support

None declared.

## Authors' contributions

Iraj Ghorbani: Study conception and planning; critical literature review.

Mozafar Khazaei: Intellectual participation in propaedeutic and/or therapeutic management of studied cases; critical manuscript review.

Hossein Kavoussi: Approval of the final version of the manuscript; effective participation in research orientation; Intellectual participation in propaedeutic and/or therapeutic management of studied cases; study conception and planning.

Ali Ebrahimi: Intellectual participation in propaedeutic and/or therapeutic management of studied cases; preparation and writing of the manuscript.

Mansour Rezaei: Data collection, analysis, and interpretation; statistical analysis.

Reza Kavoussi: Preparation and writing of the manuscript; data collection.

Kamran Mansouri: Critical literature review.

## Conflicts of interest

None declared.
